# Pharmacokinetics and pharmacodynamics of perfluoropropane after intra-venous bolus injection of perflutren lipid microsphere injection (DEFINITY®) in healthy Chinese volunteers

**DOI:** 10.1186/s40360-023-00729-z

**Published:** 2024-01-02

**Authors:** Pengfei Li, Ping Du, Jun Peng, Zhixia Zhao, Huiling Li, Weiyue Yu, Shumin Wang, Lihong Liu

**Affiliations:** 1 Department of Pharmacy, Beijing An Ding Hospital, Capital Medical University, No.5 Ankang Hutong, Xicheng District, Beijing, 100088 China; 2 Phase I Clinical Trial Unit, Beijing Chao-Yang Hospital, Capital Medical University, No.8 Gongti South Road, Chaoyang District, Beijing, 100020 China; 3 Guoke Excellence (Beijing) Medicine Technology Research Co., Ltd, No.18 Zhonghe street, Daxing District, Beijing, 100176 China; 4 Department of Pharmacy, China-Japan Friendship Hospital, No.2 Yinghuayuan East Street, Chaoyang District, Beijing, 100029 China

**Keywords:** Definity, Perfluoropropane (PFP), Pharmacokinetics, Pharmacodynamics, GC-MS, Doppler ultrasonography

## Abstract

**Background and objective:**

Definity is an ultrasound contrast agent consisting of phospholipids-encapsulated perfluoropropane (PFP), also known as perflutren, microspheres, which is initially designed to enhance echocardiographic ultrasound images. With no pharmacologic action, Definity can increase the backscatter of ultrasound resulting enhanced ultrasound signals. The objective of this study was to determine the pharmacokinetics (PKs), Pharmacodynamics (PDs) and safety of Definity in healthy male and female Chinese volunteers.

**Methods:**

A simple GC-MS method was developed and applied to simultaneously quantify PFP both in human whole blood and in expired air using Perfluorobutane (PFB) as internal standard. Meanwhile, the blood microbubble Doppler intensities were continuously monitored as companion PDs by a Doppler ultrasonography system using a non-imaging method.

**Results:**

After intravenous infusion of 10 µL/kg of PFP within 30 seconds, the mean AUC_last_ of the pharmacokinetic analysis set was 0.000653 (uL/mL)*min, the average AUC_∞_ was 0.001051 (uL/mL)*min. The main coefficient of variation of parameters were within 30%. In this trial, the blood drug concentration of female subjects was lower than that of males. Female C_max_, AUC_last_ and AUC_∞_ were lower than males’, T_max_ and t_1/2_ was close to males’, V_ss_ and CL were slightly higher than males’. The concentration of PFP in the expired air of the subject reached the maximum value 1–2 min after administration and the PFP accumulation curve in the expired air began to become flat at 9.5–11 min after administration. The PFP in the expired air at the last sampling point of most subjects was still measurable. The results of the analysis showed that female subjects had slightly more and faster PFP excretion via the lungs than males. The change of blood drug concentration in this trial was related to the change process of Doppler signal intensity. The trend of the two was close, but the peak time of blood drug concentration was slightly delayed compared with the peak time of the Doppler signal intensity. The results showed that female t_max−pd_, t_10_ was earlier than male, and women have lower AUC_pd_ than men.

**Conclusion:**

The pharmacokinetics and pharmacodynamics of Definity in blood and expired air were systematically evaluated for the first time in this study. The PK/PD analysis results of this trial showed that the change of blood concentration was related to the change process of Doppler signal intensity, the trend of the two was close and expired air are the main excretion pathways of Definity. Definity was well tolerated by all subjects in the trial.

**Trial registration:**

This study was registered on 08 December 2017 at the Chinese Clinical Trial Registry (CTR20171087).

**Supplementary Information:**

The online version contains supplementary material available at 10.1186/s40360-023-00729-z.

## Introduction

Ultrasound imaging is now in very widespread clinical use. At present, Doppler ultrasound technology has become a widely used imaging diagnostic technology, thanks to its high cost-effectiveness, safety, and non-invasive characteristics (1–2). The specificity of human tissue leads to different ultrasound echo intensities, which can provide significant image differences [3]. This real-time diagnostic test is also relatively inexpensive and highly portable compared to other imaging techniques, such as computed tomography (CT), magnetic resonance imaging (MRI), and nuclear medicine imaging. These advantages have led to the growing use of ultrasound techniques. Due to its relatively low cost and high margin of safety, ultrasound imaging is often used as a ‘first look’ or screening tool to reveal functional abnormalities or pathology [4]. Contrast enhancement is widely applied in imaging and has become a routine part of clinical x-ray, CT, and MRI. This has occurred because contrast agents in these modalities have demonstrated clinical benefits ranging from improved image quality, improved conspicuity in the detection of pathology, and improved assessments of organ structure or function [5].These same potential benefits now exist for the field of ultrasound imaging with the development of new microbubble contrast agents.

By serendipity, it was discovered that injecting saline containing small doses of tiny bubbles greatly improved the contrast of ultrasound images, and the first microbubble for diagnosis was clinically approved (6–7). The microbubble shell is composed of various materials, such as lipids, proteins, etc. Stable microbubble shells can significantly affect the ultrasound response of microbubbles [8–10]. Through further research, it was found that the microbubbles were filled with high molecular weight gases with low water solubility, such as perfluorocarbon [6, 11], which can further extend the lifespan of the microbubbles. The compressibility of the gas core enables microbubbles to respond to the contraction and expansion of the ultrasonic field, and the harmonic echoes generated by these volume oscillations are much stronger than the tissue echoes, resulting in significant ultrasound enhancement.

The basic requirements for an ideal microbubble contrast agent include absence of safety concern and appropriate acoustic response. In addition, pharmacokinetics is also an indispensable evidence for a deeply insight of mechanism and for an optimal clinical usage [12]. Definity is a new ultrasound contrast agent which has been marketed in the US, Canada and Europe. Despite the safety and efficacy of Definity have been confirmed by previous clinical studies and post-marketing experience, its PKs features including half-life, elimination and blood exposure are still unkown. Therefore, this study is planned to depict pharmacokinetic and Pharmacodynamic profiles in Chinese population using a gas chromatography tandem to mass spectrometry (GC-MS) analytical and doppler ultrasound method.

## Materials and methods

### Chemicals and reagents

Perflutren Lipid Microsphere Injection (DEFINITY®, batch number: 4696Y) was provided by China Resources Double-Crane Pharmaceutical Co. Ltd and produced by Lantheus MI Canada, Inc. Perfluoropropane (PFP, 100% purity, batch number: N40M-1602 A-2a) and perfluorobutane (PFB, 99.9% purity, batch number: N2HP-0603B-13) were purchased from Fluoro Med. Normal saline (0.9% NaCl) were purchased from Tianjin Baxter Medical Supplies Co., Ltd. All the other chemicals used in the preparation of samples were of reagent grade or better unless otherwise specified.

### Instrumentation

The GC-MS system consisted of a GC system (Shimadzu, Japan) equipped with AOC 5000 automatic sampler and a mass spectrometer (Shimadzu, Japan) with EI source. Equipment control, data and analysis were acquired and processed using the GCMS solution Version 4.42 and PAL Cycle composer Version 1.6.0.

Medasonics Versatone model D8 Doppler Ultrasound Instrument with accompanying P84 (5.3 MHz) transducer and ultrasound conduction gel suitable for human use, these have been re-wired for use at 220-240 V 50 Hz and provided with appropriate surge protector outlet strips. A package of 5 spare fuses is also included. Lenovo laptop computer running Windows 7 with study-specific acquisition/data reduction software installed and verified. Audio data will be digitized at 44,100 samples per second during subject data acquisition directly into database.

### Chromatographic conditions

For quantitative analysis, chromatographic separations were performed on GS-Gas-Pro column (60 m x 0.32 mm i.d., P/N 113–4362; Agilent, USA) and column temperature was maintained at 100℃ for the GC oven. The column flow rate was 1.0 mL/min and cleaning flow rate was 5.0 mL/min, respectively. Subject’s expired air and whole blood were added to a headspace vial with helium as the mobile phase. The injection liner was splitless glass liner with Split type and the Split ratio was 10.The injection port, interface and tester temperature was 200℃, 200℃ and 250℃, respectively. GC operating time was 10.0 min with solvent delay 4.3 min. Headspace autosampler AOC 5000 incubation and syringe temperature was kept at 80℃, 90℃, respectively. The injection volume was set at 0.5 mL for whole blood and expired air samples.

### MS conditions

Analytes were detected by mass spectrometer with an Electron Impact Ion Source (EI). The response of PFP was calculated by summing up three integral peak areas collected by m/z 169, 119, and 100 using Selective Ion Monitoring (SIM), while m/z 69 and 119 for the internal standard PFB. The response ratio of analyte and IS were used to construct the standard curve.

### Preparation of calibrators and quality control samples

Use 1L syringe to take 800 mL air and inject it in 1 L Tedlar bag; and then, use 250 µL airtight needle to precisely measure 200 µL PFB Reference Standards gas to inject into the gas bag. Knead the gas bag about 3 min and adequately mix PFB with air to take it as the internal standard stock sample for standby purpose. Prepare nine 20mL injection headspace bottles and seal them. 4 bottles are marked by “5 µL PFP” and 5 bottles are marked by “100µL PFP” for standby purpose. Open the valve of PFP standards tank and regulate air flow so that the copper tube immersed in water can produce 4～6 air bubbles every second. Insert the airtight needle in the sampling port via the gasket film (yellow face of the gasket film shall be upward). Fill the needle with air and discharge it to the air stream. Rinse at least three times. Precisely measure 5 µL PFP Reference Standards by the 25 µL airtight needle. Immediately inject the gas in the headspace bottle. Slowly roll the headspace bottle at least 30 s so that PFP gas is evenly distributed. It’s used as the Reference Standards stock sample 1, with the concentration of ~ 0.233µL/mL; 4 units in parallel. Precisely measure 100 µL PFP standards by the 250 µL airtight needle. Immediately inject the gas in the headspace bottle. Slowly roll the headspace bottle at least 30 s so that PFP gas is evenly distributed. It’s used as the Reference Standards stock sample 2, with the concentration of ~ 4.650µL/mL; 5 units in parallel. Prepare nine 20 mL injection headspace bottles; separately and precisely inject 12 mL normal saline and 2 mL blank whole blood. Seal and mark it for standby purpose. According to the precision, injected the reserve gas of reference standards of different concentrations and volumes to labeled 20mL headspace bottles. Prepared reference standards of standard curve in different concentration. Use 25 µL airtight needle to precisely measure 15 µL internal standard (PFB) prepared and add it to all headspace bottles above respectively. During sampling, pay real-time attention to the gas flow; in the whole process, sustain the number of air bubble produced basically consistent;

PFP concentration in human whole blood and expired air were detected through fully validated GC-MS with PFB as internal standard. For whole blood, the range of linearity was 0.0000310-0.0620000 µL/mL and lower limit of quantification was 0.0000310 µL/mL. For expired air, the range of linearity was 0.0000208-0.0415000 µL/mL and lower limit of quantification was 0.0000208 µL/mL.

### Whole blood samples preparation

Whole blood samples (approximately 5.0 mL per subject per time point) were collected in a heparinized Vacutainer injection syringe within 30 min before administration of perflutren lipid microsphere injection and 40s (± 10s), 1 min (± 10s), 1.5 min (± 10s), 2 min (± 10s), 2.5 min (± 10s), 3 min (± 10s), 4 min (± 10s), 5 min (± 10s), 6 min (± 10s), and 7 min (± 10s) after administration. The post-injection samples were obtained at these time points relative to the start of the injection (not the end of the injection). Immediately inject 2.0 mL of blood sample into a pre-weighed 20 mL GC headspace vial containing 12 mL of saline (with 2.0 mL of headspace removed to create a vacuum).The vials were weighed again to calculate the exact amount of blood added, and the weights were recorded in the case report form. Repeat this step with a second vial that could be used for re-analysis if required and make sure that the blood sampling tube was identified/labeled clearly and accurately. The samples were stored at RT after sample collection until analysis of PFB gas was performed.

### Exhaled air samples preparation

One minute in exhaled air samples were collected in a non-diffusing gas sampling bag immediately prior to administration of the study agent on Day 1 (baseline). the start time of administration of perflutren lipid microsphere injection was set as zero, and all expired air was collected segmentally by time intervals 0–1 min, 1–2 min, 2–3 min, 3–4 min, 4–5 min, 5-6.5 min, 6.5-8 min, 8-9.5 min, 9.5–11 min, 11-12.5 min, 12.5–14 min and 14-15.5 min. The expired air sample was transferred to an individual headspace vial from the gas sampling bag. Make sure that the sampling bags were labeled clearly and accurately and keep the samples at RT after sampling.

### Data acquisition of doppler ultrasound instrument

The arm to be used for the Doppler acquisition should be the same arm as will be used for injection of Definity. The arm should be positioned with the medial surface (i.e. palm) facing up. c. Apply ultrasound acoustic conduction gel liberally to the semicircular cavity of the P84 Doppler probe and check the gel for air bubbles. Any large bubbles or air pockets in the gel should be eliminated before continuing. The probe should be firmly in place but not so tight that it causes a reduction in the intensity of the Doppler sounds. Use one or more additional pieces of adhesive tape to secure the probe cable to the subject’s arm in a way that it will not interfere with the Definity injection. It should be possible for subjects to remain relaxed and relatively immobile for at least 25 min during data acquisition and more for preparation. It is recommended that subjects be seated or lying down with the arm in a comfortable position.

### Method validation

**Fig. 1:**
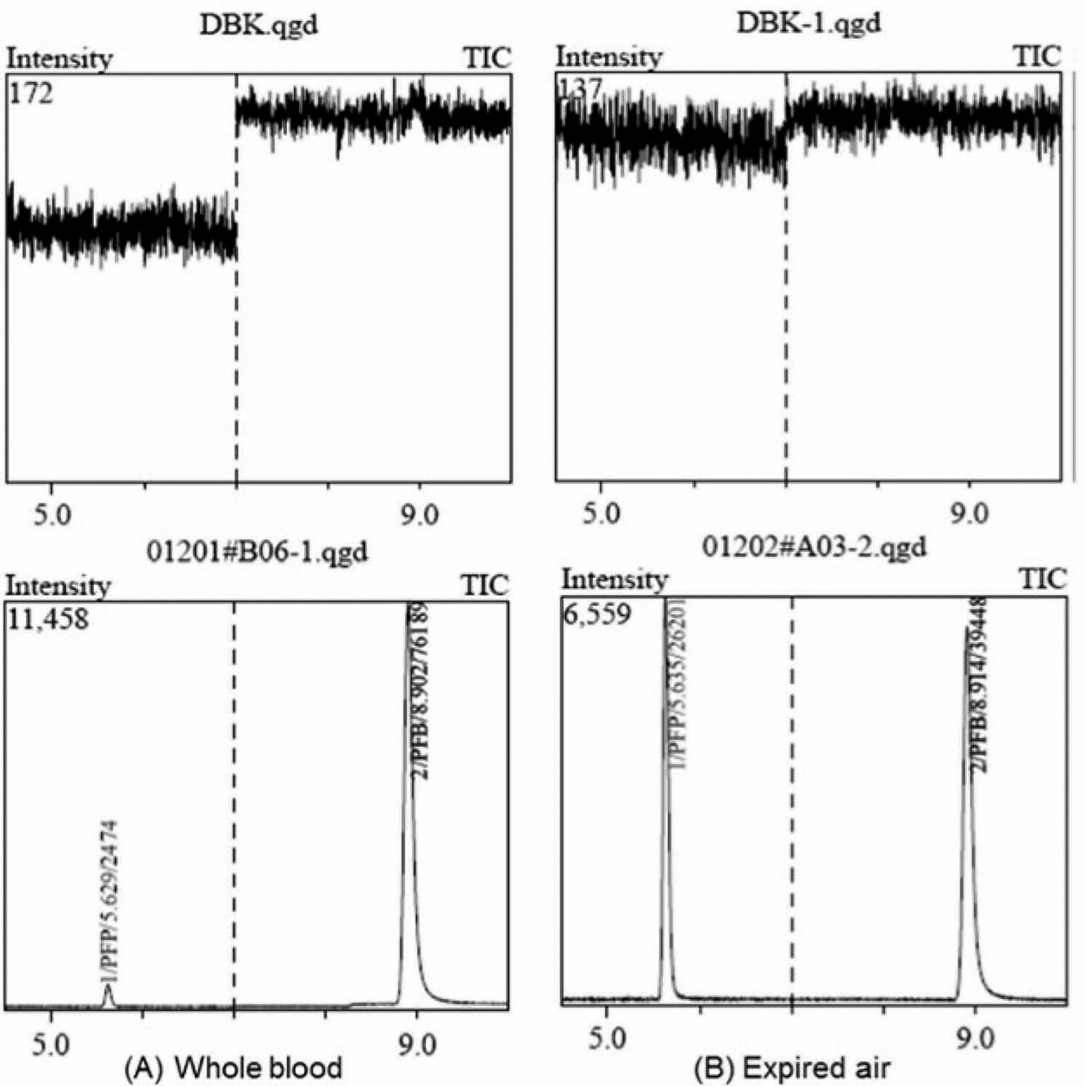
Representative SIM chromatograms for PFP (**A**) Whole blood samples (**B**) Expired air samples. After intravenous administration of PFP (10 uL/kg); Peak 1, PFP (t_R_=5.6 min); Peak 2, PFB (t_R_=8.9 min)

Complete validation of the presented method regarding system applications, selectivity, specificity, precision, accuracy, recovery, matrix effect, carryover, linearity and stability was performed in expired air and whole blood samples, according to the 9012 Guidelines for the Quantitative Analysis of Biological Samples of 2015 Chinese Pharmacopoeia. The methodology validation results displayed conform to the acceptance standard. Under the GC-MS conditions, there was no interfering peak at the elution times of the analytes and IS in the blank matrices. Examples of the typical SIM chromatograms of the analytes in whole blood and expired air samples are shown in Fig. 1, which demonstrated that PFP and PFB were well separated and the peak shapes were satisfactory. The retention times of PFP and PFB were 5.627 min, 8.999 min, respectively. This method is sensitive enough to quantitative detection of PFP and PFB.

### Clinical study

This was a Phase I, single-center, open-label, safety and pharmacokinetics study of Definity in healthy male and female Chinese volunteers. The protocol and informed consent form were approved by the ethics committee of Beijing Chao-yang Hospital, Capital Medical University (BCYH-CMU) with the ethical approval number 2016-drug-45, and all volunteers provided with informed written consent in BCYH-CMU. In this study, 2 to 4 healthy volunteers were planned to be enrolled for the pilot trial; 8 to 12 healthy volunteers are planned to be enrolled for the formal trial.4 healthy volunteers were actually enrolled in the pre-trial; 12 healthy volunteers were enrolled in the formal trial; 16 healthy volunteers had completed the trial. No healthy volunteers exited the trial. Each subject reported to the clinical unit on Day-1 to obtain baseline evaluations and admission to the unit. DEFINITY® (Perflutren Lipid Microsphere Injection) was provided in a 2mL clear vial containing 1.5mL fill volume. The Definity vial was activated prior to use with mechanical shaking device (Vialmix®). Upon activation, Definity appears as a milky white suspension. The activated product has an initial concentration of perflutren of 150 ± 100 µL/mL. All healthy volunteers received a single 10 µL/kg bolus dose of Definity on Day 0 by IV bolus injection over 30 s, followed by a 5 mL saline flush. The safety of healthy volunteers was continuously monitored within 1 h after the administration of Definity. Whole blood was collected from before administration to 7 min after the administration of Definity and expired air was collected through 15.5 min after administration. The blood sample was taken from the arm opposite to the arm that was administering the drug. It should be noted that in this study, we not only inquire about the smoking situation of healthy subjects but also use smoking detectors for breath testing to ensure that there are no smoking factors affecting this study. Additionally, during the hospitalization observation period, subjects are prohibited from entering or leaving at will and are prohibited from bringing their own food and drink; smoking, drinking, etc. are also prohibited. Please note that this strict protocol is necessary to ensure the accuracy and reliability of our research findings. PFP concentrations in blood and expired air were determined using the validated GC-MS method. Doppler ultrasound measurements were performed for generation of a relative blood microbubble Doppler intensity-time curve for comparison to blood concentrations of PFP. Ultrasound measurements were acquired using a commercial ultrasound system with appropriate transducer operating at a center frequency of 3.5 MHz. Ultrasound monitoring began at least 5 min prior to dosing and continued until 20 min post dosing. Within 72(± 24) hours after Definity administration, healthy volunteers received a safety follow-up by telephone call to collect adverse events and concomitant medication information and conduct necessary review. This study was confirmatory study, and dose selection was based on recommended IV maximum single dose established in foreign phase 1 and phase 1 echocardiography and abdominal ultrasound efficacy trials. The recommended single bolus injection maxium dose in the product description is 10 µL/kg. Besides, according to clinical trial approval of NMPA, it is required to perform single dose PK study with the recommended maxium dose. Therefore, 10 µL/kg was used as the study dose. Certificate of Analysis (COA) of PFP concentration for drugs from Lot 4696Y, reported in December 8, 2016, is provided in the reference materials. The COA lists the results of 6 test vials for “PFP volume” and averages the results to get 152 µL (95% confidence interval; 113µL to 190µL) PFP per milliliter of emulsion after activation.

### Statistical analysis

All statistical analysis were conducted by using SAS version 9.4 (SAS Institute, Inc., Cary, NC).Sample size, arithmetic mean, geometric mean, standard deviation, coefficient of variation, median, minimum, and maximum were used to indicate the analysis results of pharmacokinetic parameters. Standard descriptive summaries included the case number (n), mean, median, standard deviation (SD), minimum, and maximum for continuous variables; for categorical variables the number of cases and percentage of different available data are calculated. All statistical tests used two-sided test, α = 0.05, unless otherwise specified and Cl used 95% two-sided Cl.

## Results and discussion

### Demographic study and safety evaluation

In this trial,16 subjects were enrolled in pre-trial and formal trial, all of whom were Asians, 1 Manchu, 15 Hans, with an average age of 26.7 years old (SD = 4.85 years old, range 19 ~ 36 years old). There were 12 male subjects (75%), and 4 female subjects (25%), with an average body weight of 60.43 kg (SD = 7.759 kg, range 50.1 ~ 72.4 kg), and an average height of 169.09 cm (SD = 9.083 cm, range 151.5 ~ 183.5 cm). Average body mass index (BMI) was 21.06 kg/m^2^ (SD = 1.560 kg/m^2^, range 19.1 ~ 23.9 kg/m^2^). The comparison of height, weight, and BMI between male and female healthy volunteers is as follows: male average height is 173.0 cm (SD = 6.3 cm, range 160.0 ~ 183.5 cm) vs. female average height of 157.4 cm (SD = 4.8 cm, range 151.5-160.3 cm), male average weight is 63.4 kg (SD = 6.5 kg, range 53.3 ~ 72.4 kg) vs. female average weight of 51.5 kg (SD = 1.4 kg, range 50.1 ~ 52.7 kg). Male average BMI is 21.2 kg/m^2^ (SD = 1.7 kg/m^2^, range 19.1 ~ 13.9 kg/m^2^) vs. female average BMI of 20.8 kg/m^2^ (SD = 1.1 kg/m^2^, range 19.8 ~ 21.9 kg/m^2^). Safety evaluation indicators include such results as adverse events, serious adverse events, laboratory tests (Hematology, blood biochemistry, Urinalysis, coagulation function, pregnancy examination), vital signs (pulse, systolic and diastolic blood pressure, respiratory rate, body temperature, and transcutaneous oxygen saturation), 12-lead electrocardiogram, and physical examination. There were no serious adverse events and no adverse events that led to the suspension or cessation of drug use. In the study, the investigators completed the trial drug preparation and intravenous administration for each subject in strict accordance with the trial protocol. The preparation process, administration method, dose, and administration time did not deviate from the trial protocol. Compliance of the subject is good. Therefore, 4 subjects in the pre-trial and 12 subjects in the formal trial received a single intravenous injection of 10 µL/kg DEFINITY®, with good compliance.

### Pharmacokinetic study

**Fig. 2:**
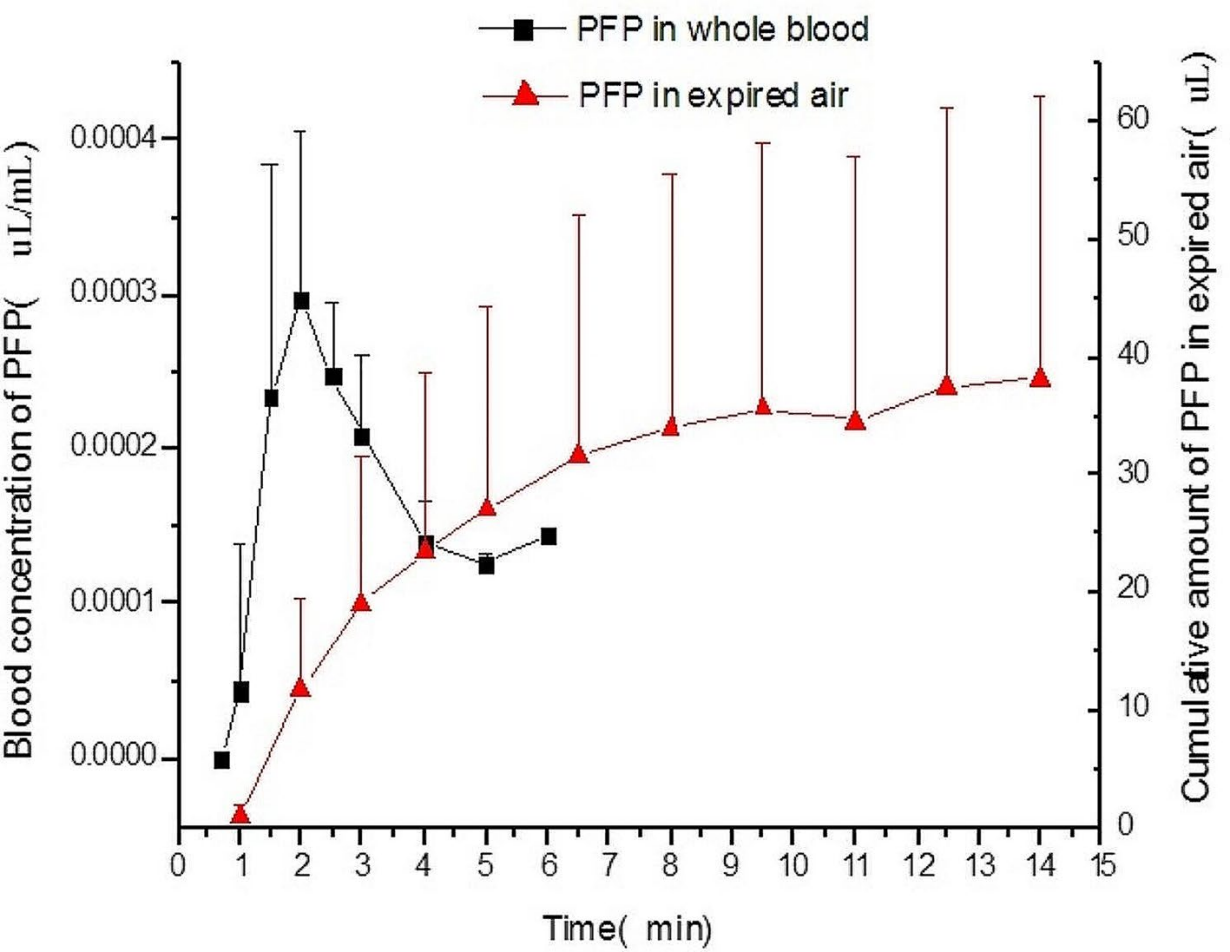
The mean blood concentration-time profiles and the exhaled air concentration-time profiles of PFP

The GC-MS methods which have been developed and validated were successfully applied to the pharmacokinetic study, and the quantitative range proved to be reasonable. The mean blood concentration-time profiles and the exhaled air concentration-time profiles of Definity in different humans are presented in Fig. 2, and the corresponding pharmacokinetic parameters in the blood calculated by non-compartmental analysis are summarized in Table 1 ~ 2, the corresponding pharmacokinetic parameters in expired air calculated by non-compartmental analysis are summarized in Table 3 ~ 4. The following statistics were calculated based on the PFP concentrations in blood and the amounts in expired air: number of cases (n), arithmetic mean, geometric mean, standard deviation, coefficient of variation (CV), median, minimum and maximum values. Plots of the mean PFP concentration – time curve, including linear and semi-logarithmic linear graph. At the same time, based on the PFP concentration data of each subject measured in the trial, individual drug concentration-time curves were drawn, including linear and semi-logarithmic linear graph, so as to fully reflect the characteristics of the drug in terms of absorption, distribution, and metabolism in the human body. Phoenix WinNonlin 8.0 was used to assess the pharmacokinetic properties of PFP in blood and expired air through non-compartmental methods: peak blood levels of PFP (C_max_), time of peak blood levels (t_max_), area under the concentration-time curve at the last measurable concentration (AUC_last_), area under the concentration-time curve (AUC) extrapolated to infinity, terminal elimination half-life (t_½_), clearance (CL), and apparent volume of distribution (V_ss_). Cumulative excretion of PFP in expired air and clearance of PFP via the lungs (CL_lung_) are measured.

**Table 1:** Calculation result of PFP pharmacokinetic parameters in the blood

Pharmacokinetic Parameters	n	Arithmetic mean(SD)	Coefficient of variation of arithmetic mean (%)	Median	Min – Max	Geometric mean	Coefficient of variation of geometric mean (%)
C_max_ (uL/mL)	12	0.00034475 (0.000132329)	38.4	0.0002864	0.0001840–0.0005811	0.00032293	38.8
T_max_ (min)	12	2.06 (0.454)	22.1	2.0000	1.5–3.0	2.01	22.1
AUC_last_ ((uL/mL)*min)	12	0.000653 (0.0003084)	47.3	0.00063	0.00024–0.00134	0.000586	53.2
AUC_∞_ ((uL/mL)*min)	10	0.001051 (0.0003247)	30.9	0.001015	0.00068–0.00178	0.001012	29.0
t_1/2_ (min)	10	1.68 (0.326)	19.4	1.6500	1.2–2.3	1.65	19.2
CL (L/hr)	10	5395.362 (1306.935)	24.2	5669.095	3062.01–7061.51	5225.103	28.6
V_ss_ (L)	10	314.812 (89.522)	28.4	318.90	192.68–467.41	303.006	30.2

*Note*: For the calculation of CL and Vss, the PFP dose is calculated by substituting 152 uL/mL. 152uL/mL is the mean value of the “PFP volume” test results for the 6 samples of the 4696Y batch of drugs on December 8, 2016

**Table 2:** PFP pharmacokinetic parameters in the blood by gender

Pharmacokinetic parameters	Gender	n	Arithmetic mean(standard deviation)	Arithmetic coefficient of variation (%)	Median	Min – Max	Geometric mean	Geometric coefficient of variation (%)
C_max_ (uL/mL)	Male	8	0.00035852 (0.000134325)	37.5	0.0003376	0.0001840–0.0005811	0.00033626	40.4
	Female	4	0.00031719 (0.000143485)	45.2	0.00024774	0.0002410–0.0005323	0.00029785	40.3
T_max_ (min)	Male	8	2.08 (0.501)	24.1	2.00	1.5–3.0	2.02	23.9
	Female	4	2.03 (0.411)	20.3	2.05	1.5–2.5	1.99	21.4
AUC_last_ ((uL/mL)*min)	Male	8	0.000691 (0.0003440)	49.8	0.000745	0.00024–0.00134	0.00061	61.3
	Female	4	0.000575 (0.0002464)	42.8	0.000505	0.00036–0.00093	0.000541	41.1
AUC_∞_ ((uL/mL)*min)	Male	6	0.001143 (0.0003185)	27.9	0.00105	0.00090–0.00178	0.001114	24.2
	Female	4	0.000913 (0.0003235)	35.4	0.00079	0.00068–0.00139	0.000876	32.5
t_1/2_ (min)	Male	6	1.63 (0.308)	18.8	1.60	1.2–2.1	1.61	19.3
	Female	4	1.75 (0.387)	22.1	1.65	1.4–2.3	1.72	21.2
CL (L/hr)	Male	6	5289.987 (1205.885)	22.8	5624.865	3062.01–6418.42	5148.346	27.5
	Female	4	5553.425 (1626.382)	29.3	5928.34	3295.51–7061.51	5342.39	34.6
V_ss_ (L)	Male	6	308.275 (97.835)	31.7	283.3	214.34–467.41	296.335	31.1
	Female	4	324.618 (88.747)	27.3	361.225	192.68–383.34	313.297	33.4

After intravenous administration of 10 µL/kg of the activated product over 30 s, In this trial, the blood drug concentration of the subject after administration reached a peak at 2.06 min after the start of administration, and the last measurable time point was 6 min after the start of administration, and the elimination half-life was 1.68 min. PFP measurable blood collection points in the blood of subjects 01205 and 01207 were too few, resulting in less than 3 measurable points in the elimination phase therefore; the elimination rate constant λz could not be calculated, t_1/2_ (= 0.693/λz), AUC_∞_(= AUC_last_+AUC_last/λz_), CL(= PFP dose / AUC_∞_)) and Vss (= CL/λz).As shown in the Table 1, the mean AUC_last_ of the pharmacokinetic analysis set was 0.000653 (uL/mL)*min (median = 0.000630 (uL/mL)*min, range 0.00024 ~ 0.00134 (uL/mL)*min),the average AUC_∞_ was 0.001051 (uL/mL)*min (median = 0.001015 (uL/mL)*min, range 0.00068 ~ 0.00178 (uL/mL)*min). Except for the coefficient of variation of C_max_ and AUC_last_ exceeding 30%, the coefficient of variation of other parameters were within 30%.

After administration, the blood drug concentration of female subjects was lower than that of males. Female C_max_, AUC_last_ were lower than males’, T_max_ and t_1/2_ was close to males’, V_ss_ and CL were slightly higher than males’. The female subject reached the maximum value 2 min after the start of administration, and the last measurable PFP concentration in the blood was from 3 to 4 min after administration, and the last measurable PFP concentration was 0.0001108 uL/mL to 0.0001558 uL/mL. The PFP peak time in women’s blood was consistent with the overall results. The final detectable concentration time point was slightly different from the overall result. It may be related to that only 4 female subjects were included in this trial, and the male to female ratio was 2:1. Table 2 summarizes the PFP pharmacokinetic parameters in blood by gender. The results showed that women’s C_max_, AUC_last_ and AUC_∞_ were lower than men, with ratios of 0.88, 0.83 and 0.80, respectively. Female T_max_ and t_1/2_ was close to males’, with a difference of 0.05 and 0.12 min, respectively. V_ss_ and CL were also close to males’, both the ratios of female V_ss_ and CL to males were approximately 1.05. It showed that women’s drug exposure was slightly lower than that of men, while T_max_, the process of distribution and clearance were close to that of men.

**Table 3:** Calculation result of PFP pharmacokinetic parameters in expired air

Pharmacokinetic Parameters	n	Arithmetic mean(SD)	Coefficient of variation of arithmetic mean (%)	Median	Min – max	Geometric mean	Coefficient of variation of geometric mean (%)
Ae_0 − 5_ (uL)	12	30.455 (18.668)	61.3	24.18	8.932–63.669	25.25	73.4
Ae_0 − 15.5_ (uL)	12	42.041 (24.631)	58.6	34.09	13.887–83.565	35.25	70.9
Ae_0 − tlast_ (uL)	11	25.039 (15.246)	60.9	21.05	7.607–50.920	20.70	74.9
Ae_∞_(uL)	12	44.997 (26.010)	57.8	36.89	15.401–90.499	38.01	68.7
CL_lung_ (L/hr)	12	3203.8 (2407.95)	75.2	2416.50	982–7978	2501.50	84.2
PFP excretion rate^1^ (%)	12	47.89 (30.523)	63.7	34.80	15.1–103.7	39.42	74.3
PFP excretion rate^2^ (%)	12	64.45 (41.069)	63.7	46.85	20.4–139.5	53.06	74.3
PFP excretion rate^3^ (%)	12	38.33 (24.436)	63.8	27.85	12.1–83.0	31.54	74.3

*Note*: PFP excretion rate: Cumulative excretion volume of PFP (0-15.5 min) in the expired air ÷ PFP dose

In this trial, the concentration of PFP in the expired air of the subject reached the maximum value 1–2 min after administration. The slope of the PFP accumulation curve in the expired air increased 1–2 min after administration, and the PFP accumulation curve in the expired air began to become flat at 9.5–11 min after administration. The PFP in the expired air at the last sampling point of most subjects was still measurable. As shown in the Table 4, Female subjects had higher concentrations of PFP in expired air at each time point after administration than men. The average Ae_0 − 5_ of the subjects was 30.455 uL, the average Ae_0 − 15.5_ was 44.997 uL, the average CL_lung_ was 3203.8 L/hr, and the average PFP excretion rate was 47.89%. The results of the subgroup analysis showed that the ratios of female Ae_0 − 5_, Ae_0 − 15.5_, Ae_∞_, CL_lung_, and PFP excretion rate (calculated at 152 uL/mL) to males’ were 1.86, 1.74, 1.73, 1.72, and 2.14, respectively, indicating that female subjects had slightly more and faster PFP excretion via the lungs than males.

**Table 4:** PFP pharmacokinetic parameters in expired air by gender

Pharmacokinetic parameters	Gender	n	Arithmetic mean (standard deviation)	Arithmetic coefficient of variation (%)	Median	Minimum value-Maximum value	Geometric mean	Geometric coefficient of variation (%)
Ae_0 − 5_ (uL)	Male	8	23.690 (13.448)	56.8	19.397	8.932–48.388	20.601	61.9
	Female	4	43.984 (22.131)	50.3	49.278	13.711–63.669	37.941	79.7
Ae_0 − 15.5_ (uL)	Male	8	33.715 (19.543)	58	28.893	13.887–63.435	29.171	62.7
	Female	4	58.692 (27.906)	47.5	65.828	19.549–83.565	51.457	73.9
Ae_0 − tlast_ (uL)	Male	7	18.883 (11.045)	58.5	15.564	7.607–36.744	16.29	64.4
	Female	4	35.812 (16.958)	47.4	40.083	12.163–50.920	31.488	72.6
Ae_∞_(uL)	Male	8	36.156 (20.445)	56.5	30.631	15.401–67.628	31.556	60.1
	Female	4	62.680 (29.664)	47.3	69.417	21.385–90.499	55.161	72.2
CL_lung_ (L/hr)	Male	8	2583.1 (2137.52)	82.7	1856	982–7318	2037.9	79.7
	Female	4	4445.3 (2741.71)	61.7	4122	1559–7978	3769.1	78.6
PFP excretion rate^1^ (%)	Male	8	34.74 (18.641)	53.7	30.55	15.1–65.3	30.6	58.5
	Female	4	74.20 (34.942)	47.1	83.7	25.7–103.7	65.43	71.4
PFP excretion rate^2^ (%)	Male	8	46.75 (25.088)	53.7	41.1	20.4–87.9	41.18	58.5
	Female	4	99.85 (47.006)	47.1	112.65	34.6–139.5	88.05	71.3
PFP excretion rate^3^(%)	Male	8	27.79 (14.927)	53.7	24.4	12.1–52.3	24.47	58.5
	Female	4	59.40 (27.950)	47.1	67.0	20.6–83.0	52.39	71.3

### Pharmacodynamics study

Curve of ultrasonic signal amplitude changes along time of each subject were drawn in accordance with the ultrasonic signal data (minus the baseline value) obtained to indicate the influence of the injected drug on the ultrasonic signal of the body. Time to maximum signal (t_max−pd_), time from max signal to 10% max signal (t_10_), and Definity-related enhanced curve (AUC_pd_) were calculated based on the data. Based on the actual values of Doppler signal measurement, Phoenix WinNonlin 8.0 was used to assess t_max−pd_ and AUC_pd_ through non-compartmental methods. For the negative Definity-related enhanced data points, they were set 0 when calculating AUC_pd_. In order to reduce the influence of random noise, t_10_ was calculated based on the average value of the actual values measured within 30s according to the time interval of 30s. Case number, arithmetic mean, geometric mean, standard deviation, coefficient of variation, median, minimum, and maximum were used to indicate the analysis results of pharmacodynamic parameters. As shown in Table 5, the pharmacodynamic analysis had a mean t_max−pd_ of 125.7 s (median = 70.5 s, range 63–615 s) and a mean t_10_ of 359.0 s (median = 354.0 s, range 48 ~ 618 s). The arithmetic coefficient of variation of t_max−pd_ reached 125.3%, suggesting that there may be abnormal values. Use the Dixon test to determine the outliers of the Doppler ultrasound signal parameters, and it was found that 01209, 01210 subjects had abnormal values. These two subjects were excluded from the pharmacodynamic analysis for sensitivity analysis. The remaining 10 subjects had a mean t_max−pd_ of 71.2 s (median = 69.0 s, range 63–87 s) and a mean t_10_ of 405.0 s (median = 357.0 s, range 288 ~ 618 s).

**Table 5:** Calculation result of Doppler ultrasonic signal parameters

Pharmacodynamic parameters	n	Arithmetic mean (SD)	Coefficient of variation of arithmetic mean (%)	Median	Min – max	Geometric mean	Coefficient of variation of geometric mean (%)
t_max−pd_ (sec)	12	125.7 (157.44)	125.3	70.5	63–615	91.7	74.3
t_10_ (sec)	12	359.0 (152.60)	42.5	354	48–618	312.7	73.8
AUC_pd_ (dB*sec)	12	1076.33 (496.293)	46.1	879.3	541.5–2161.2	988.93	43.6
t_max−pd_ (sec)	10	71.2 (7.44)	10.4	69	63–87	70.9	10.1
t_10_ (sec)	10	405.0 (113.57)	28	357	288–618	392.5	26.1
AUC_pd_ (dB*sec)	10	1050.29 (543.757)	51.8	846.2	541.5–2161.2	950.62	47.3

*Note*: t_10_ is the time from the maximum signal to the 10% maximum signal. t_10_ is based on 30s interval ultrasound signal data. For data points where the Definity correlation enhancement is negative, the valude is set to 0 when calculating AUC_pd_

**Fig. 3:**
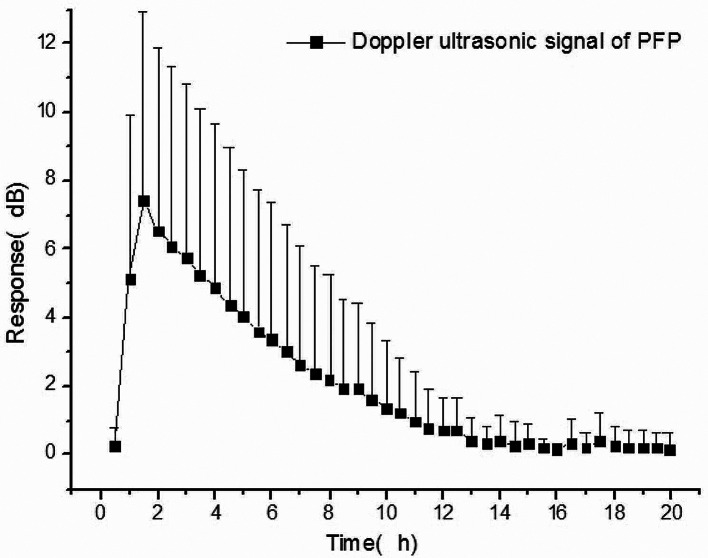
The relative enhancement-time map of the blood concentration of the Doppler signal

At the same time, based on the blood drug concentration and Doppler ultrasound signal 6s interval data, the relative enhancement-time map of individual blood drug concentration and Doppler signal was drawn. See Fig. 3 for details. The relative enhancement-time map of the blood concentration of the individual and the Doppler signal showed that the change of blood concentration was related to the change process of Doppler signal intensity, and the trend of the two was close. The peak time of blood drug concentration was slightly delayed compared with the peak time of the Doppler signal. As the concentration of PFP in the blood decreased, the Doppler ultrasound signal also weakened. When the Doppler signal intensity of the subject decreased from the maximum signal to 10% of maximum signal, the blood drug concentration had passed 3 to 7 half-lives. In addition, the Doppler signal intensity could still be measured 6 min after the start of administration, after the blood drug concentration was below the lower limit of quantitation. The results of the Doppler ultrasound signal parameters by gender in the pharmacodynamic analysis are shown in Table 6. The results showed that female t_max−pd_, t_10_ was earlier than male, and the difference was 44.5 and 84 s, respectively. Women have lower AUC_pd_ than men, with a ratio of 0.67.

**Table 6:** Doppler ultrasonic signal parameters by gender

pharmacodynamics parameters	Gender	n	Arithmetic mean (standard deviation)	Arithmetic coefficient of variation(%)	Median	Minimum – maximum value	Geometric mean	Geometric coefficient of variation(%)
t_max−pd_ (sec)	Male	8	140.5 (191.88)	136.6	72	63–615	94.5	89.0
	Female	4	96.0 (56.79)	59.2	70	63–181	86.4	52.8
t_10_ (sec)	Male	8	387.0 (178.34)	46.1	378	48–618	321.3	96.0
	Female	4	303.0 (70.06)	23.1	321	210–360	296.3	25.5
AUC_pd_ (dB*sec)	Male	8	1210.14 (546.032)	45.1	1063.45	684.4–2161.2	1113.66	44.9
	Female	4	808.70 (253.364)	31.3	774.35	541.5–1144.6	779.81	31.9

### Relationship of definity dose-lung clearance-boold exposure-doppler response

**Fig. 4:**
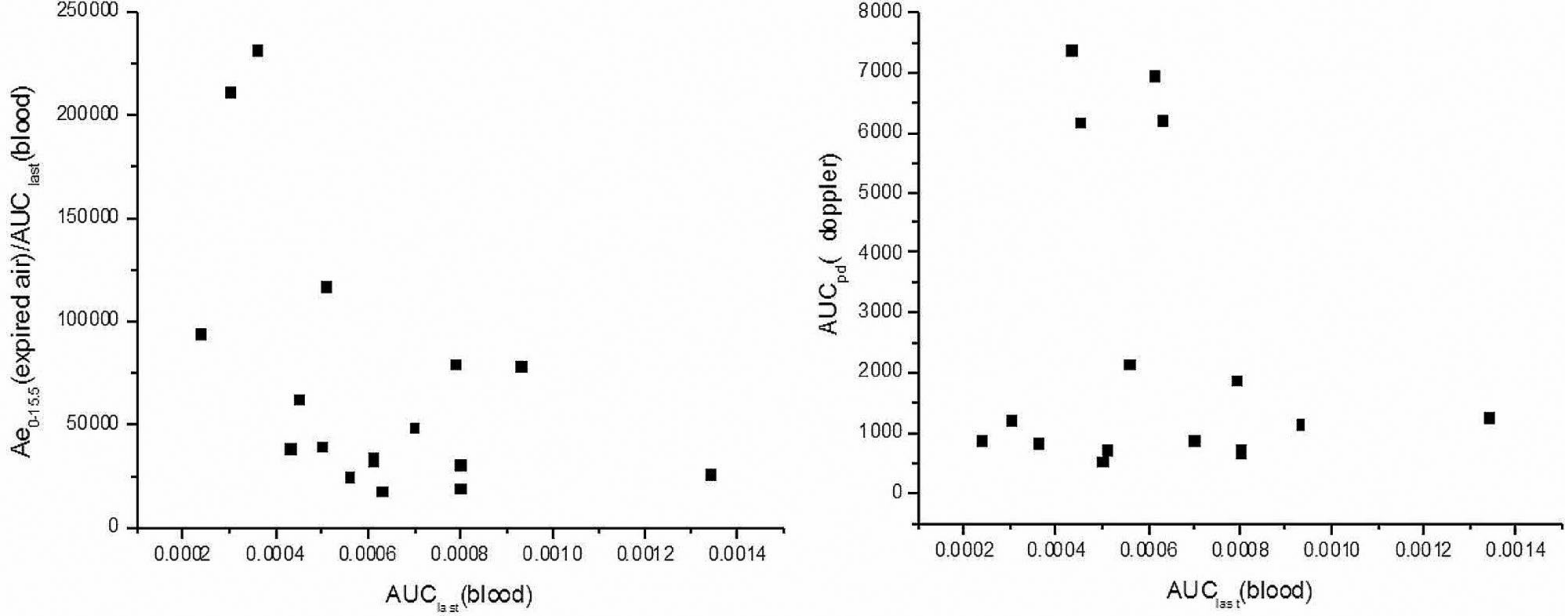
The relationship between blood, expired air and doppler signal

PFP, as the key ingredient of Definity, is an inert gas playing important role in keeping the bubble stable in blood, resulting in a prolonged contrast enhancement in ultrasound imaging. Due to no metabolism has been identified after dosing, excretion from lung by expired gas was supposed to be the only route of PFP elimination in vivo. Thus, the results is reasonable that the more the lung clearance, the less the blood exposure, as the Fig. 4 showed. However, the relationship between boold exposure and doppler response showed that acoustic intensity (max signal, AUCpd) did not change much as PK parameters changed (C_max_, AUC_last_ and AUC_∞_). This phenomenon can be explained by theory of non-linear saturation dynamics, which could be deduced by the results that undetectable PFP level in blood in the terminal phase can still lead to considerable intensive doppler response. Of cause, the most convincing evidence is to enlarge the dosing range in further in-depth experiments.

The most interesting thing need to note here is the lagged peak time from the end of injection for both blood concentration and acoustic intensity, which is superficially exhibited as a 1.5-minute absorption phase in blood concentration curve. This is considered as abnormality because compound dissolved so instantly and the circulation velocity was so fast that chemical drug generally attain the peak concentration in blood at the end time of injection. However, PFP was injected by enveloped bubbles, some of the larger bubbles would like to be entrapped in pulmonary capillary where only smaller bubbles can pass through. After all PFP bubbles were reformed to a smaller size, the pulmonary capillary is no longer a barrier for PFP bubble to return to the heart and body circulation. In other word, for PFP disposition in vivo, lung is not only the organ of excretion but also a site of reprocessing.

### Comprehensive discussion on Pharmacokinetics and Safety

The pharmacokinetics of PFP in humans have not been reported. In a recent study by Yang et al., a direct determination method for PFP in dog whole blood was established, and the pharmacokinetics of PFP suspension after intravenous infusion of microcapsules was studied [13]. The results showed that PFP in dogs still exhibits the characteristics of reaching its peak earlier (T_max_ is 30 s) and elimination faster (t_1/2_ is 44 s), but due to interspecies differences, there is no comparability between drug exposure and pharmacokinetic participation. In previous studies, we found that PFB, a similar contrast agent to PFP, peaked quickly after intravenous administration in the Chinese population [14]. (T_max_ was 1.5 ± 0.5 min and 1.9 ± 1.4 min for the 0.12 µL and 0.60 µL MB/kg b.w. dose groups, respectively). PFB was also rapidly eliminated (t_1⁄2_ was 2.7 ± 0.7 min and 17.0 ± 7.7 min for the 0.12 µL and 0.60 µL MB/kg b.w. dose groups, respectively). The shape of the estimation curve suggested a biphasic estimation profile. Landmark KE studied the pharmacokinetics of PFB in healthy volunteers and patients with reduced lung diffusivity after intravenous and continuous infusion of sorrazole in Caucasians [15]. Blood concentrations of PFB decreased biphasically, with an elimination half-life ranging from 30 to 45 min. The AUC values in patients with increased gas diffusion were significantly larger than those in healthy volunteers. The excess kinetics of PFB quantification varied, with t_1⁄2_ ranging from 28 to 111 min. Compared with PFB, PFP in this study reached its peak earlier and eliminated faster. PFP in blood and exhaled gas was lower than the quantification limit after 6 and 14 min, respectively. Both PFB and PFP showed significant individual differences in blood and exhaled gas. In the above human studies, PFB and PFP did not cause clinically significant changes in laboratory tests, vital signs, blood oxygen saturation, and electrocardiogram, and both showed good human safety.

## Conclusion

Definity is an ultrasound contrast agent based on microbubbles containing PFP gas. In the present study, we reported a robust GC-MS assay has been developed which is sensitive and selective, shows good linearity of response and high precision for fast and accurate determination of PFP in the whole boold and expired air. The Doppler ultrasonography system was used to assess the blood microbubble Doppler intensity. The methods were successfully applied to the investigation of the Pharmacokinetic and Pharmacodynamics of PFP. As shown in Fig. 2 ~ 4, the trend of blood concentration and Doppler signal intensity was similar, the Doppler response is enhanced as the whole blood concentration increases, while the higher whole blood exposure, the lower expired gas output. The t_max_ of whole blood drug concentration and doppler response were both about 2 min, it was speculated that intravenous administration and Doppler signal collection were done in the same arm, while blood collection was for the other arm, and PFP microbubbles were distributed and circulated through blood and tissues after entering the blood, thus achieving Doppler signal enhancement.

In summary, the change of blood drug concentration in this trial was related to the change process of Doppler signal intensity. The trend of the two was close, but the peak time of blood drug concentration was slightly delayed compared with the peak time of the Doppler signal intensity. In addition, when the blood drug concentration was lower than the lower limit of quantitation, the Doppler signal intensity could still be measured. Assessment of the Pharmacokinetic profile of Definity in air in the current trial, the slope of the PFP accumulation curve in the expired air increased after administration, and the PFP accumulation curve in the expired air began to be flat at 9.5–11 min. After iv administration, expired air are the main excretion pathways of Definity. Definity was well tolerated by all subjects in the trial. There were no SAEs or AEs that led to withdrawal from the study. The AEs that occurred were mainly Hematology laboratory tests with mildly abnormal results that returned to normal during follow-up.

### Electronic supplementary material

Below is the link to the electronic supplementary material.

**Supplementary Material 1:** Schedule and dose of Subject Administration

**Supplementary Material 2:** CONSORT-2010-Checklist

## Data Availability

The datasets during the current study are available from the corresponding author on reasonable request after the main results have been published, as long as it corresponds with the local rules and regulations for data sharing.
