# Supplementary material for: Pharmacokinetics and pharmacodynamics of perfluoropropane after intra-venous bolus injection of perflutren lipid microsphere injection (DEFINITY®) in healthy Chinese volunteers
Source: BMC Pharmacol Toxicol. 2024 Jan 2;25:6. doi: 10.1186/s40360-023-00729-z (PMC10763448; doi:10.1186/s40360-023-00729-z)
Supplement: Supplementary file 1 — Supplementary Material 1: Schedule and dose of Subject Administration [file 40360_2023_729_MOESM1_ESM.docx]

**Appendix 1** Schedule and dose of Subject Administration

| Subjects’ enrollment No. | Date of administration | Administration started from | Administration ended by | End time of saline injection | Route of administration | Dosage （μL/kg） | Dosing volume（ml） | PFP dosage ^1^（uL） | PFP dosage ^2^（uL） | PFP dosage ^3^（uL） |
| --- | --- | --- | --- | --- | --- | --- | --- | --- | --- | --- |
| 01201 | 2017-11-17 | 08:00:00 | 08:00:30 | 08:01:00 | Intravenous injection | 10 | 0.60 | 91.2 | 67.8 | 114 |
| 01202 | 2017-11-17 | 09:00:00 | 09:00:30 | 09:01:00 | Intravenous injection | 10 | 0.60 | 91.2 | 67.8 | 114 |
| 01203 | 2018-02-05 | 08:00:00 | 08:00:30 | 08:00:48 | Intravenous injection | 10 | 0.71 | 107.92 | 80.23 | 134.9 |
| 01204 | 2018-02-05 | 09:00:00 | 09:00:30 | 09:01:00 | Intravenous injection | 10 | 0.54 | 82.08 | 61.02 | 102.6 |
| 01205 | 2018-03-12 | 08:00:00 | 08:00:30 | 08:01:00 | Intravenous injection | 10 | 0.63 | 95.76 | 71.19 | 119.7 |
| 01206 | 2018-03-12 | 09:00:00 | 09:00:30 | 09:00:59 | Intravenous injection | 10 | 0.66 | 100.32 | 74.58 | 125.4 |
| 01207 | 2018-03-12 | 10:00:00 | 10:00:30 | 10:00:59 | Intravenous injection | 10 | 0.71 | 107.92 | 80.23 | 134.9 |
| 01208 | 2018-03-20 | 08:00:00 | 08:00:30 | 08:01:00 | Intravenous injection | 10 | 0.58 | 88.16 | 65.54 | 110.2 |
| 01209 | 2018-03-20 | 09:00:00 | 09:00:30 | 09:00:59 | Intravenous injection | 10 | 0.59 | 89.68 | 66.67 | 112.1 |
| 01210 | 2018-03-20 | 10:00:00 | 10:00:30 | 10:00:58 | Intravenous injection | 10 | 0.50 | 76 | 56.5 | 95 |
| 01211 | 2018-03-21 | 08:00:00 | 08:00:30 | 08:01:00 | Intravenous injection | 10 | 0.50 | 76 | 56.5 | 95 |
| 01212 | 2018-03-21 | 09:00:00 | 09:00:30 | 09:01:00 | Intravenous injection | 10 | 0.53 | 80.56 | 59.89 | 100.7 |
| 01213 | 2018-03-21 | 10:00:00 | 10:00:30 | 10:01:00 | Intravenous injection | 10 | 0.54 | 82.08 | 61.02 | 102.6 |
| 01214 | 2018-03-22 | 08:00:00 | 08:00:30 | 08:01:00 | Intravenous injection | 10 | 0.53 | 80.56 | 59.89 | 100.7 |
| 01215 | 2018-03-22 | 09:00:00 | 09:00:30 | 09:01:00 | Intravenous injection | 10 | 0.63 | 95.76 | 71.19 | 119.7 |
| 01216 | 2018-03-22 | 10:00:00 | 10:00:30 | 10:01:00 | Intravenous injection | 10 | 0.71 | 107.92 | 80.23 | 134.9 |

^1^PFP dose: Subject weight (kg) × 10 (μL/kg) ×152 (μL/mL). The actual dosage volume is taken into the calculation.

^2^PFP dose: Subject weight (kg) × 10 (μL/kg) ×113 (μL/mL). The actual dosage volume is taken into the calculation.

^3^PFP dose: Subject weight (kg) × 10 (μL/kg) ×190 (μL/mL). The actual dosage volume is taken into the calculation.

Data source: statistical analysis report, attached Listing 16.2.5.
